# Enhanced detection with spectral imaging fluorescence microscopy reveals tissue- and cell-type-specific compartmentalization of surface-modified polystyrene nanoparticles

**DOI:** 10.1186/s12951-016-0210-0

**Published:** 2016-07-07

**Authors:** Kata Kenesei, Kumarasamy Murali, Árpád Czéh, Jordi Piella, Victor Puntes, Emília Madarász

**Affiliations:** School of PhD Studies, Semmelweis University, Üllői Street 26, Budapest, 1085 Hungary; Institute of Experimental Medicine, Hungarian Academy of Sciences, Szigony Street 43, Budapest, 1083 Hungary; Soft Flow Hungary Kft., Kedves u. 20, Pecs, 7628 Hungary; Catalan Institute of Nanoscience and Nanotechnology, Campus UAB, Bellaterra, 08193 Barcelona, Spain

**Keywords:** Spectral imaging fluorescence microscopy, Polystyrene nanoparticle, Nanoparticle surface, Toxicity, Macrophage, In vivo distribution

## Abstract

**Background:**

Precisely targeted nanoparticle delivery is critically important for therapeutic applications. However, our knowledge on how the distinct physical and chemical properties of nanoparticles determine tissue penetration through physiological barriers, accumulation in specific cells and tissues, and clearance from selected organs has remained rather limited. In the recent study, spectral imaging fluorescence microscopy was exploited for precise and rapid monitoring of tissue- and cell-type-specific distribution of fluorescent polystyrene nanoparticles with chemically distinct surface compositions.

**Methods:**

Fluorescent polystyrene nanoparticles with 50–90 nm diameter and with carboxylated- or polyethylene glycol-modified (PEGylated) surfaces were delivered into adult male and pregnant female mice with a single intravenous injection. The precise anatomical distribution of the particles was investigated by confocal microscopy after a short-term (5 min) or long-term (4 days) distribution period. In order to distinguish particle-fluorescence from tissue autofluorescence and to enhance the detection-efficiency, fluorescence spectral detection was applied during image acquisition and a post hoc full spectrum analysis was performed on the final images.

**Results:**

Spectral imaging fluorescence microscopy allowed distinguishing particle-fluorescence from tissue-fluorescence in all examined organs (brain, kidney, liver, spleen and placenta) in NP-treated slice preparations. In short-time distribution following in vivo NP-administration, all organs contained carboxylated-nanoparticles, while PEGylated-nanoparticles were not detected in the brain and the placenta. Importantly, nanoparticles were not found in any embryonic tissues or in the barrier-protected brain parenchyma. Four days after the administration, particles were completely cleared from both the brain and the placenta, while PEGylated-, but not carboxylated-nanoparticles, were stuck in the kidney glomerular interstitium. In the spleen, macrophages accumulated large amount of carboxylated and PEGylated nanoparticles, with detectable redistribution from the marginal zone to the white pulp during the 4-day survival period.

**Conclusions:**

Spectral imaging fluorescence microscopy allowed detecting the tissue- and cell-type-specific accumulation and barrier-penetration of polystyrene nanoparticles with equal size but chemically distinct surfaces. The data revealed that polystyrene nanoparticles are retained by the reticuloendothelial system regardless of surface functionalization. Taken together with the increasing production and use of nanoparticles, the results highlight the necessity of long-term distribution studies to estimate the potential health-risks implanted by tissue-specific nanoparticle accumulation and clearance.

**Electronic supplementary material:**

The online version of this article (doi:10.1186/s12951-016-0210-0) contains supplementary material, which is available to authorized users.

## Background

Nanoparticles (NPs) are increasingly popular tools with widespread industrial, medical and every-day applications. While the continuous progress in NP production is appealing, the escalation of environmental NP pollution raises serious health concerns. Despite of world-wide efforts, our understanding on the penetration, distribution, potential accumulation and clearance of various NPs in the living body is far from complete.

Several imaging approaches have already been used to visualize nanoparticles in vitro or in vivo [1]. Some studies investigated NP distribution at the whole-body level by using magnetic resonance imaging, computed tomography, positron emission tomography, or radiolabeling techniques [2–4]; whereas other reports focused on the subcellular localization of NPs by exploiting transmission or scanning electron microscopy [5, 6]. Relatively few studies attempted to follow the in vivo distribution of distinct types of NPs at the tissue and cellular levels [3, 7, 8]. This approach would be important from a medical perspective, because specific tissues and cells may be differentially involved in pathophysiological responses to nanoparticle exposure. Moreover, NP-aided drug delivery seeks to target certain cell types in selected organs, while must avoid loading others in order to reduce unwanted side-effects [9–11]. To achieve targeted NP distribution, it is pivotal to understand the impact of the physical and chemical parameters of NPs on their tissue- and cell-type-specific accumulation. Yet the availability of high-throughput imaging modalities to compare the distribution of different NPs, is rather limited.

Fluorescence microscopy is generally the method-of-choice to monitor the cellular and tissue-level distribution of biologically-relevant fluorescent materials, including nanoparticles [12]. However, visualization of fluorescent NPs even with high-resolution confocal microscopy has been notoriously difficult, because of their size (typically being between 1 and 100 nm) is below Abbe’s diffraction limit of ~250 nm. Therefore, fluorochrome-functionalized NPs can only be detected if they passively aggregate in body fluids or at biological interfaces (e.g. on the blood vessel wall or on the cell surface) [13], or if they are actively taken up by cells and are concentrated within endocytotic vesicles or lysosomes [14–16]. Moreover, the detection of fluorescent NPs is further hindered by the high autofluorescence of biological samples [12], which does not allow visualizing small aggregates due to the low signal-to-noise ratio. Increasing fluorescent dye concentration on the NP surface may lead to enhanced cytotoxicity, whereas encapsulation (in “core–shell” nanoparticles) limits dye concentration per particle, thereby reducing NP fluorescence intensity [17, 18]. These constraints stressed the importance of applying new imaging approaches in the studies on tissue- and cell-type-specific NP distribution, and prompted us to exploit spectral detection and spectral analysis with the aim to overcome the limitations caused by low NP fluorescence *versus* high tissue autofluorescence.

The in vivo distribution of NPs is influenced by several physical and chemical parameters including size, shape, core material and surface composition [19]. Importantly, NPs readily adsorb various chemical substances from their environments due to the highly reactive surface [20, 21]. The composition and thickness of adsorbed layers (the so-called corona) depends on the chemical properties of both the NP surface and the environment [22–24]. Because the corona governs the interaction of NPs with biological structures, it plays a decisive role in the tissue- and cell-type-specific NP distribution [25, 26]. Moreover, as a result of chemical exchange reactions, the corona is expected to change with time even within the same tissue environment [27, 28]. While NP surfaces are ultimately functionalized by the actual environment, this process can be regulated by changing the surface charge of NPs or by coating the NP with chemically less reactive, hydrophilic polymers [29]. Polyethylene glycol (PEG) polymers with different oligomer-numbers and linear or branching chains have been widely used to reduce the chemical reactivity of surfaces [30]. Accordingly, protein adsorption by NPs could be reduced by PEGylation, and PEG-coating was shown to inhibit the cellular uptake of NPs [31–33], as well. In vivo studies demonstrated that PEGylated nanoparticles remained longer in the circulation due to their reduced attachment to vessel walls and cell surfaces [34, 35]. These findings together suggested that NPs displaying different adsorption-characteristics will show different tissue-, and cell-type-specific integration.

To investigate the impact of molecular surface characteristics on the in vivo tissue penetration and accumulation of otherwise identical NPs, we followed the fate of non-toxic polystyrene NPs with carboxylated or PEGylated surfaces by spectral imaging fluorescence microscopy. Spectral imaging has been used for localization of quantum dots before [1, 36]. The object of the study was to show that spectral imaging is a valuable tool to study the biodistribution and subcellular localization of fluorescently labeled NPs with broader emission bandwidths as well.

## Results

### In vitro characterization of polystyrene nanoparticles

Polystyrene nanoparticles core-labelled with fluorescent dyes and surface coated with either carboxyl groups (PS-COOH) or PEG (PS-PEG) were used throughout this study. The physical and chemical parameters of particles including size, aggregation properties, and protein adsorption were thoroughly analyzed. These parameters were determined in distinct inorganic or biological environments, including solutions used during particle handling and solutions that mimic the characteristics of body fluids.

**Fig. 1:**
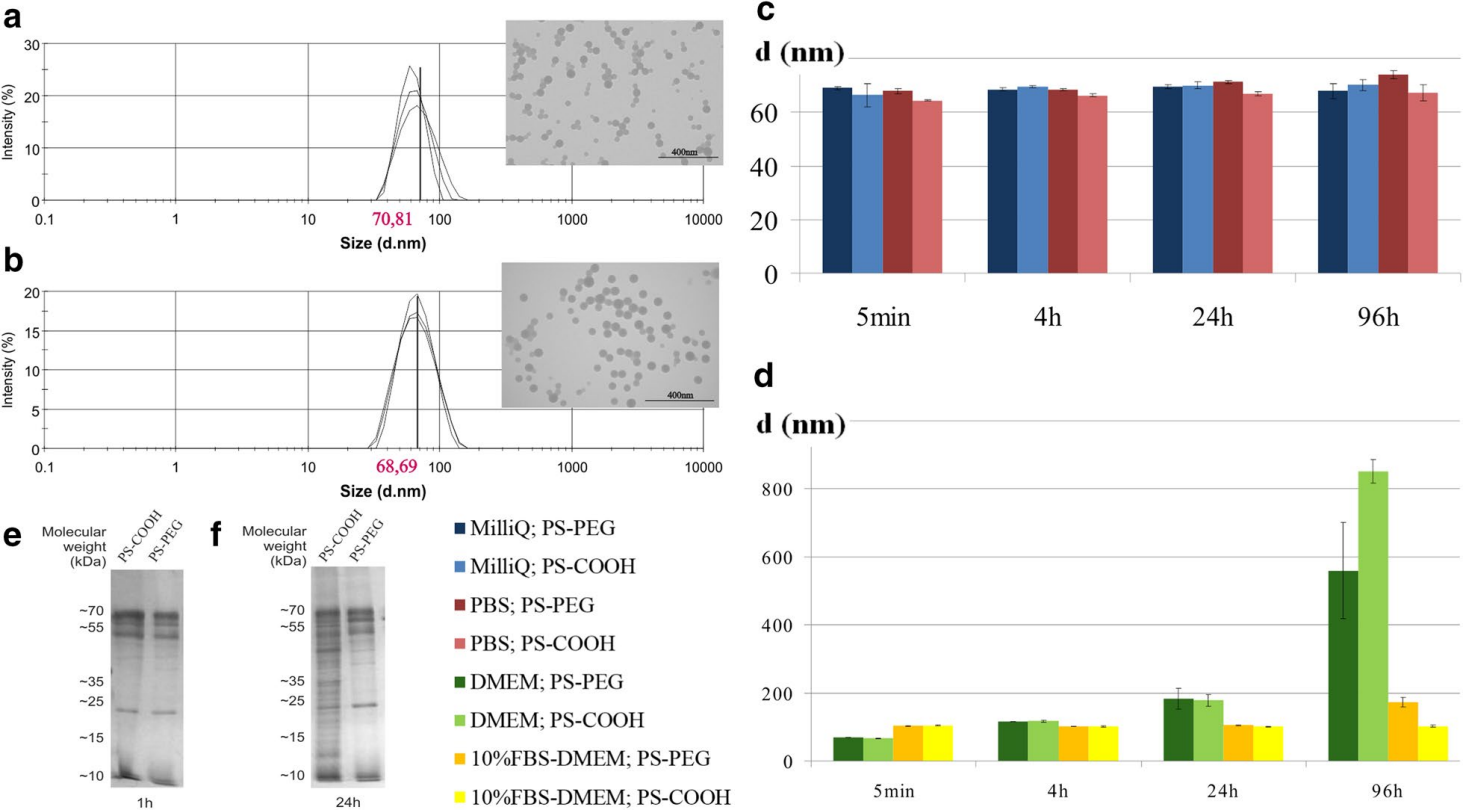
Physical-chemical characterization of polystyrene NPs. Intensity weighted size distribution of carboxylated (**a**) and PEGylated (**b**) polystyrene nanoparticles measured by dynamic light scattering. Representative TEM images of the particles are shown in the top right panels of each DLS plot, *scale bars* represent 400 nm.** c**,** d** Aggregation–agglomeration properties and corona thickening of polystyrene nanoparticles was measured by DLS as a change in hydrodynamic size. Particles were incubated in inorganic (**c**) or biological solutions (**d**) for 96 h, and the size distribution was monitored. *Color codes* of samples are shown in the figure. Data are presented as mean ± standard deviation (n = 3). Particles showed no aggregation in distilled water or in PBS during the 96-h assay period (**c**). In contrast a time-dependent, heavy particle aggregation was found in serum-free DMEM (d). Incubation of nanoparticles with 10 % FBS also evoked an immediate size increase, but prevented large-scale aggregation (**d**). SDS-PAGE analysis confirmed the adsorption of serum proteins to both PS-COOH and PS-PEG nanoparticles after 1 h incubation in 10 % serum containing MEM (e) and verified reduced protein adsorption of PEG-coated nanoparticles after 24 h incubation (**f**)

Dynamic light scattering (DLS) measurements verified the similar size of PS-COOH and PS-PEG NPs (Fig. 1a, b): 70.81 ± 21.09 and 68.69 ± 18.68 nm for PS-COOH and PS-PEG, respectively; and showed no aggregation of particles in distilled water. Transmission electron microscopic (TEM) images showed slight agglomeration of dried particles (Fig. 1a, b, insert).

The zeta potential of particles measured by DLS in distilled water, showed significant differences: −42.1 ± 0.9 mV for PS-COOH and −28.5 ± 1.8 mV for PS-PEG NPs. According to hydrodynamic particle size distribution, the nanoparticles did not aggregate in distilled water or in phosphate-buffered saline (PBS) during a 96-h assay period (Fig. 1c), indicating that the ionic strength of organic material-free physiological saline did not induce aggregation of PS-COOH and PS-PEG nanoparticles. In contrast, a time-dependent, heavy particle aggregation of both NPs was found in serum-free Dulbecco’s modified Eagle’s cell culture medium (DMEM), which represents an ionic strength similar to PBS, but contains various organic compounds including glucose, amino acids, vitamins and non-peptide hormones (Fig. 1d). In DMEM, a moderate increase in NP size was observed after 4 h, which elevated robustly by the end of the 96-h incubation [(hydrodynamic diameters for PS-COOH: 66.40 ± 0.82 nm (5 min), 116.90 ± 2.10 nm (4 h), 178.47 ± 17.39 nm (24 h), 851.77 ± 34.27 nm (96 h); for PS-PEG: 68.55 ± 0.45 nm (5 min), 115.93 ± 0.60 nm (4 h), 182.07 ± 30.53 nm (24 h), 559.67 ± 141.11 nm (96 h)]. The kinetics of particle-size enlargement is consistent with an immediate deposition of material on particle surfaces and a large-scale aggregation thereafter. The data showed that PEG-coating reduced the aggregation, an effect which was evident after long-term incubation (Fig. 1d).

The incubation of nanoparticles with 10 % fetal bovine serum also evoked an immediate size increase, but prevented large-scale NP aggregation during long-term treatment (Fig. 1d). The observation indicated that serum components were immediately adsorbed by particle surfaces, but instead of cross-linking particles, the protein corona could stabilize the suspension of the dispersed particles. Additional electrophoresis data further verified the rapid adsorption of proteins to both PS-COOH and PS-PEG nanoparticles (Fig. 1e). PEG-coated nanoparticles, however, exhibited reduced protein adsorption, which was evident after 24-h incubation (Fig. 1f) suggesting that PEGylation makes nanoparticles less prone to interactions with the environment. (Raw data of characterization experiments is shown in the Additional file 1).

Physicochemical characterization demonstrated that both, protein adsorption on nanoparticle surfaces and NP aggregation are influenced by the chemical milieu and by the type of surface-coating of NPs.

### Spectral imaging fluorescence microscopy visualizes in vivo distribution of polystyrene nanoparticles

Previous experiments demonstrated that fresh PS-COOH or PS-PEG nanoparticle preparations did not have toxic effects on several mouse neural cell lines, even when applied at a very high concentration (up to 125 µg/ml) [33]. In accordance with the in vitro observations, single intravenous injection of 33.3 µg/ml PS-COOH or PS-PEG nanoparticles did not cause detectable physiological or behavioral changes.

**Fig. 2:**
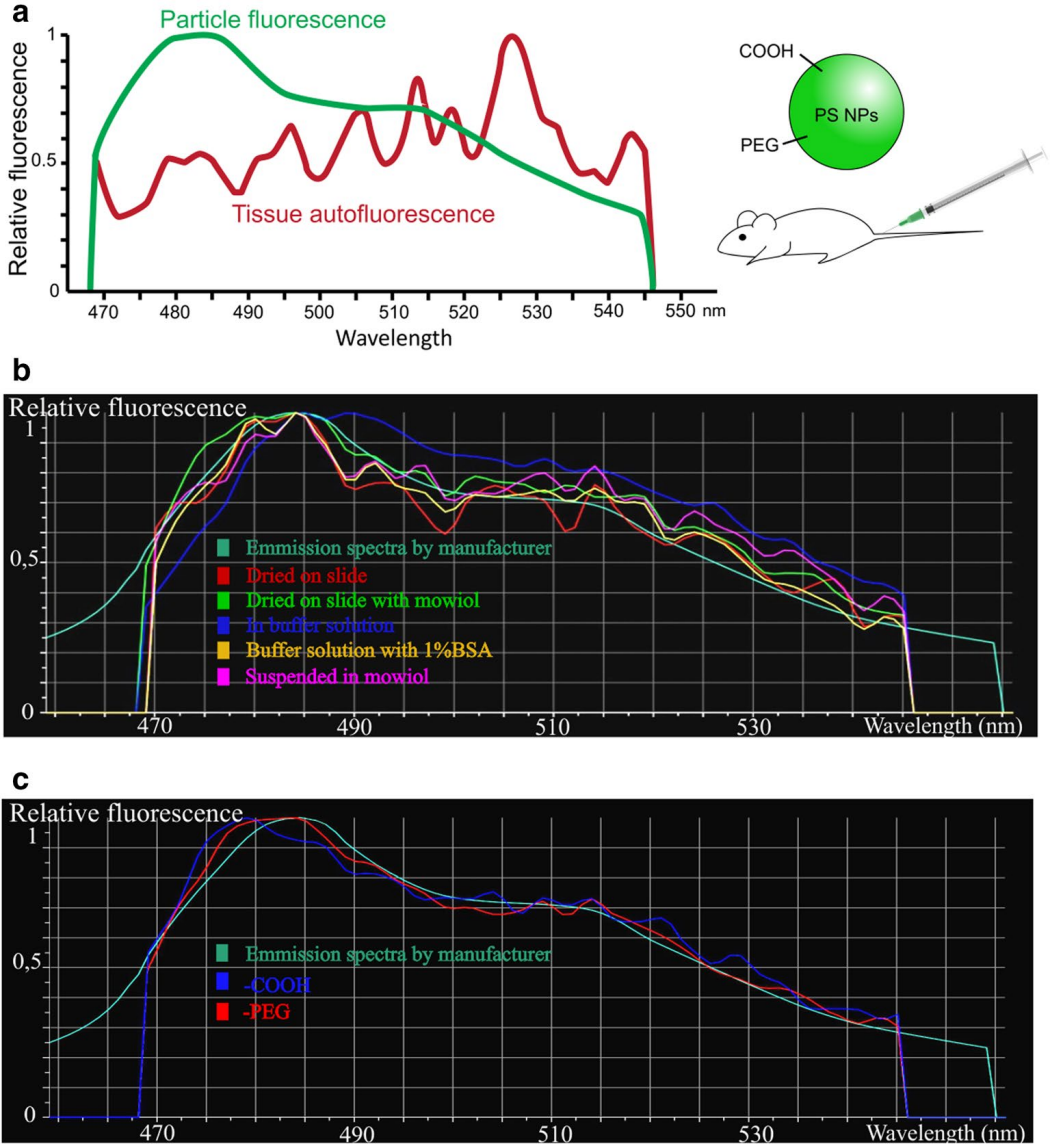
Spectral imaging fluorescence microscopy and full spectrum analysis visualizes in vivo distribution of polystyrene nanoparticles.** a** Summary of the spectral analysis of particle-fluorescence (*green*) versus tissue-autofluorescene (*red*) after a single injection of COOH- or PEG-coated polystyrene nanoparticles.** b**,** c** Emission spectra of nanoparticles measured by spectral imaging fluorescence microscopy. Specimens were excited at 457 nm and the emitted light was detected in a wavelength range from 468 to 548 nm, with a spectral resolution of 2.5 nm. Spectra of polystyrene nanoparticles did not change in different conditions (shown as *color-codes* in **b**) or after functionalization (**c**) compared to the native spectrum provided by the manufacturer (represented by the aquamarine *color-code* in the figures)

To monitor the in vivo distribution of PS-COOH and PS-PEG polystyrene nanoparticles, we developed a spectral imaging fluorescence microscopy based method, which could overcome the limitations of nanoparticle detection caused by the high autofluorescence of native tissues, and could allow analyzing larger samples in less time, compared to electron microscopic approaches. Using a multidetector array confocal arrangement [Nikon A1R Confocal Laser Microscope System equipped with a spectral detector unit (Nikon, Shinjuku, Tokyo, Japan)], fluorescence intensity was recorded at defined wavelengths throughout the investigated spectral range, from 468 to 548 nm, with a spectral resolution of 2.5 nm. This acquisition approach combined with post hoc spectrum analysis selectively visualized nanoparticles in various tissues (Fig. 2a). We found that the highest signal-to-noise ratio was achieved by illuminating the tissue-embedded fluorescent nanoparticles at 457 nm excitation wavelength (Additional file 2).

Using the optimized imaging settings, the fluorescence spectra of PS-COOH and PS-PEG NPs were measured in various environments including PBS; PBS complemented with 1 % bovine serum albumin (BSA); mounting medium (Mowiol); and seeded onto fixed tissue slices. The measured spectra of particles showed parity with the spectrum provided by the manufacturer and did not vary substantially under different conditions (Fig. 2b). Furthermore, the different surface coating had no direct effect on the NP fluorescence, PS-COOH and PS-PEG nanoparticles displayed identical fluorescence spectra (Fig. 2c). For spectral analysis, the autofluorescence of particle-free, fixed tissue-sections was used as a negative control (Additional file 3), whereas the fluorescence of NPs seeded on control tissue sections served as positive controls.

### Tissue- and cell-type-specific distribution of PS-COOH and PS-PEG nanoparticles

To evaluate short-term and the long-term distribution of NPs, mice were sacrificed either 5 min after a single tail-vein injection, or after a 4-day long survival period. In general, all investigated organs including the brain, kidney, liver, placenta and the spleen showed different amounts of nanoparticles after a single intravenous injection, and displayed distinct clearing after the 4-day post-injection period (Table 1).

**Table 1:** Biodistribution of polystyrene nanoparticles after intravenous injection. Summary of the distribution of differently functionalized polystyrene NPs after intravenous injection into mice. Organs were excised 5 min or 4 days after particles administration

	Brain	Placenta	Kidney	Liver	Spleen	Embryonic tissue
PS-COOH						
5 min	+	+	+	+	+	−
4 days	−	−	−	+	+	−
PS-PEG						
5 min	−	−	+	+	+	−
4 days	−	−	+	+	+	−

*Five minutes after nanoparticle injection*, high abundance of both PS-COOH and PS-PEG NPs were found in organs involved in the elimination of toxic products from the body, such as the kidney, liver and the spleen. In organs protected by complex physiological barriers, such as the brain and the placenta, high density of PS-COOH NPs was revealed, while PS-PEG NPs were not present (Table 1).

**Fig. 3:**
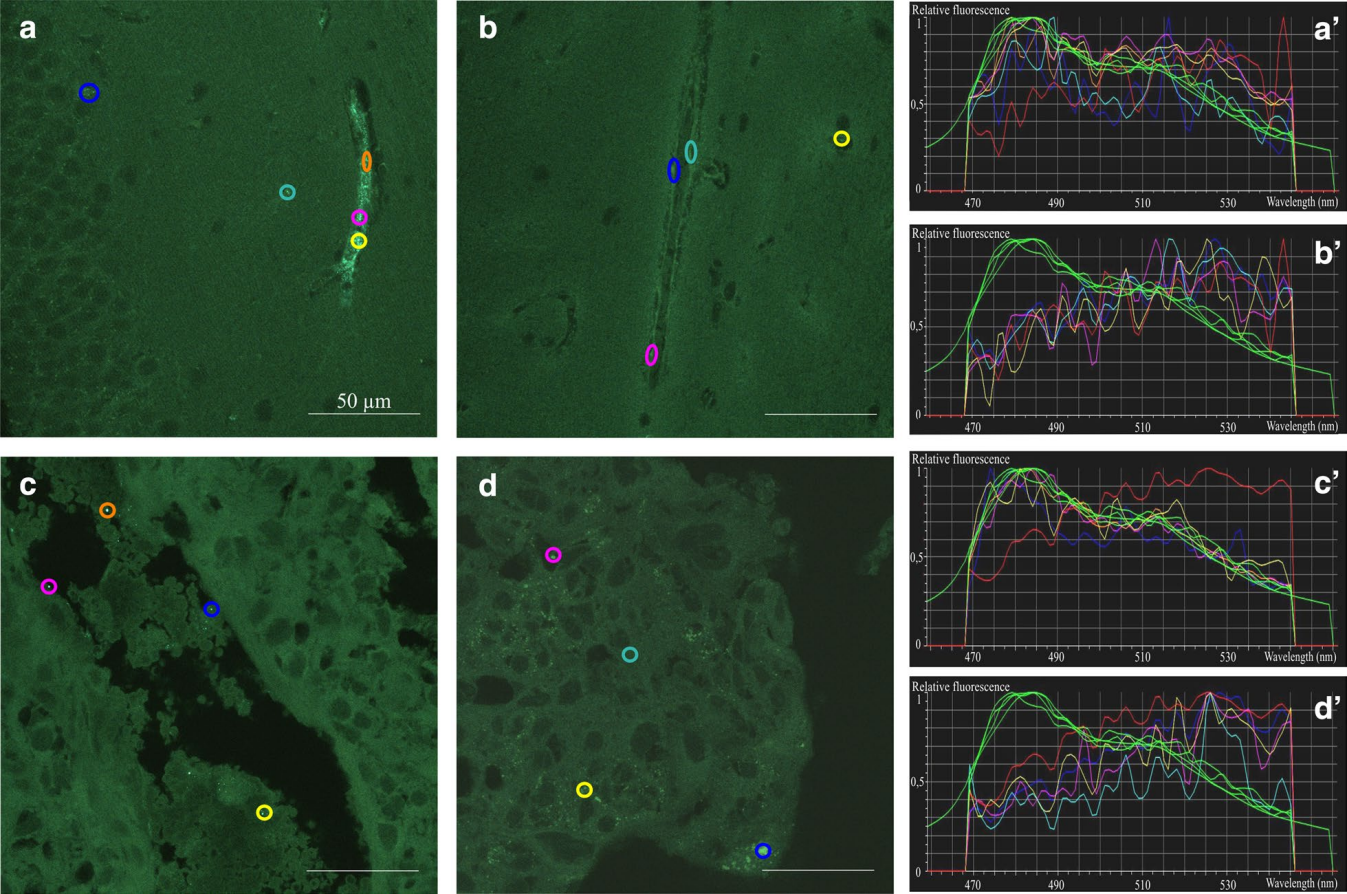
Distribution of polystyrene NPs in the brain and the placenta 5 min after systemic exposure. Spectral images of tissue sections from the mouse brain (**a**,** b**) and placenta (**c**,** d**) 5 min after injection of PS-COOH (**a**,** c**) or PS-PEG (**b**,** d**) nanoparticles into the tail vein.** a′**–**d′**: spectrum profiles of ROIs in the corresponding images. The spectrum of each ROI is marked with the same* color* as it is delineated in the microscopic image. *Green curves* represent particle fluorescence (positive controls); *red curve* represents tissue autofluorescence (negative control). *Scale bars* 50 µm

The high-resolution of confocal microscopy combined with spectral imaging enabled more detailed analysis of regional and cellular distribution of nanoparticles in various tissues. Notably, aggregated PS-COOH NPs were concentrated in large vessels and capillaries within the brain, whereas the parenchyma was largely devoid of NPs (Fig. 3a, b; Additional file 4). In the placenta, PS-COOH NPs, but not PS-PEG NPs were seen in the lacunas (Fig. 3c, d), and importantly, neither type of nanoparticles was found in embryonic tissues (Additional file 5), indicating a proper placental barrier function.

**Fig. 4:**
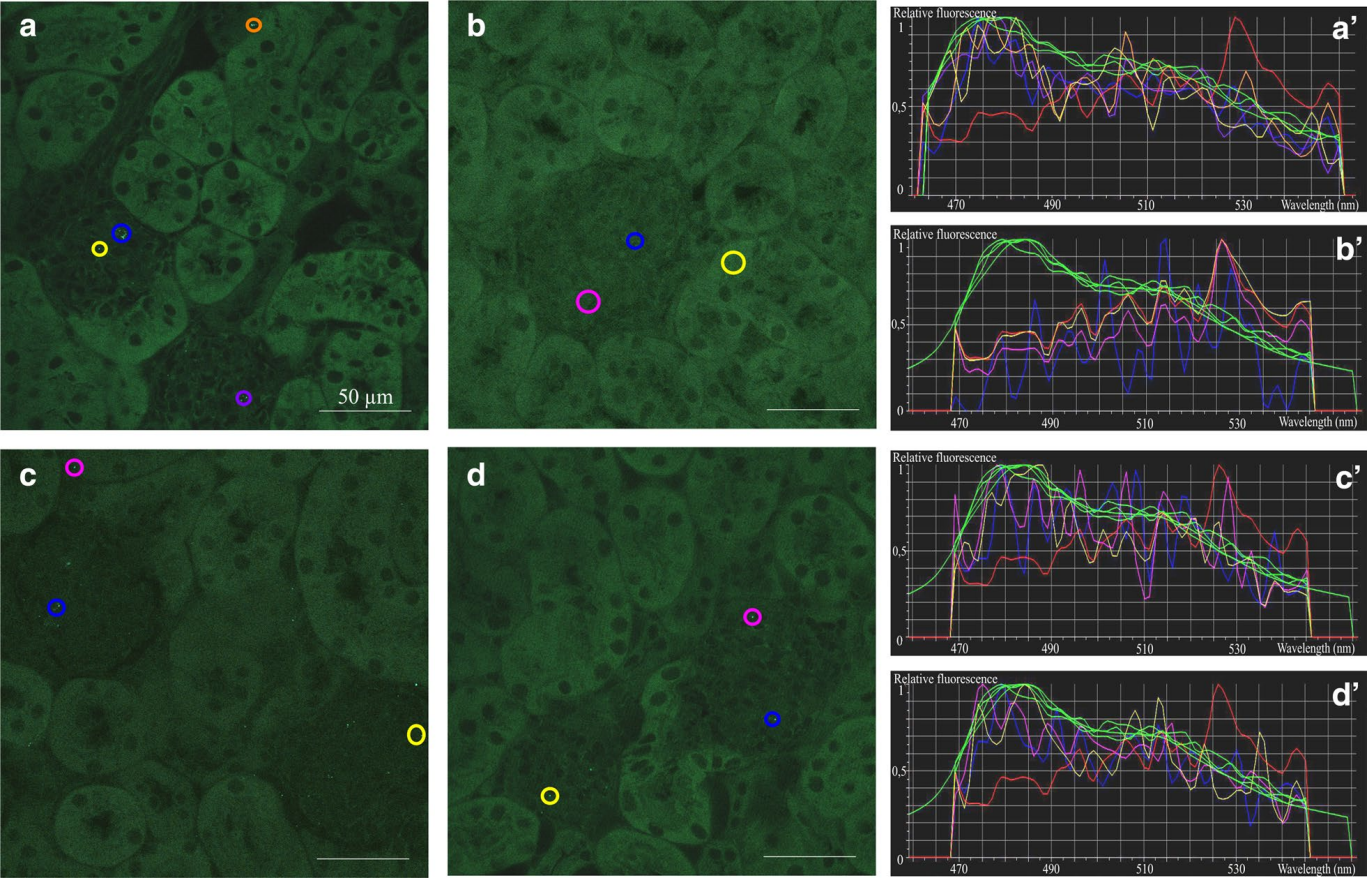
Distribution and accumulation of polystyrene nanoparticles in the kidney. Spectral images of adult mouse kidney sections 5 min (**a**,** c**) and 4 days (**b**,** d**) after injection of PS-COOH (**a**,** b**) or PS_PEG (**c**,** d**) nanoparticles through the tail vein.** a′**–**d′**: spectrum profiles of ROIs in the corresponding images. The spectrum of each ROI is marked with the *same color* as it is delineated in the microscopic image. *Green curves* represent particle fluorescence (positive controls); *red curve* represents tissue autofluorescence (negative control). *Scale bars* 50 µm

**Fig. 5:**
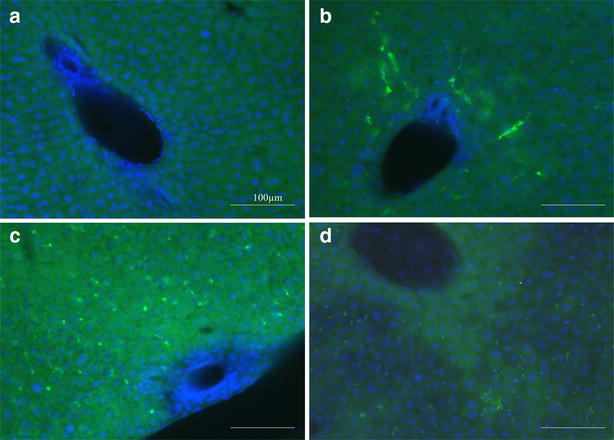
Accumulation of polystyrene nanoparticles in the liver. Traditional fluorescence microscopic images of sections made from livers of non-treated (**a**) and NP-injected (**b**,** c**,** d**) adult mice. Animals were sacrificed 5 min (**b**) and 4 days (**c**) after intravenous injection of PS-COOH and 4 days after injection of PS-PEG (**d**) polystyrene nanoparticles. Particle fluorescence is shown in *green*, and bisbenzimide nuclear staining in *blue*. *Scale bars* 100 µm

**Fig. 6:**
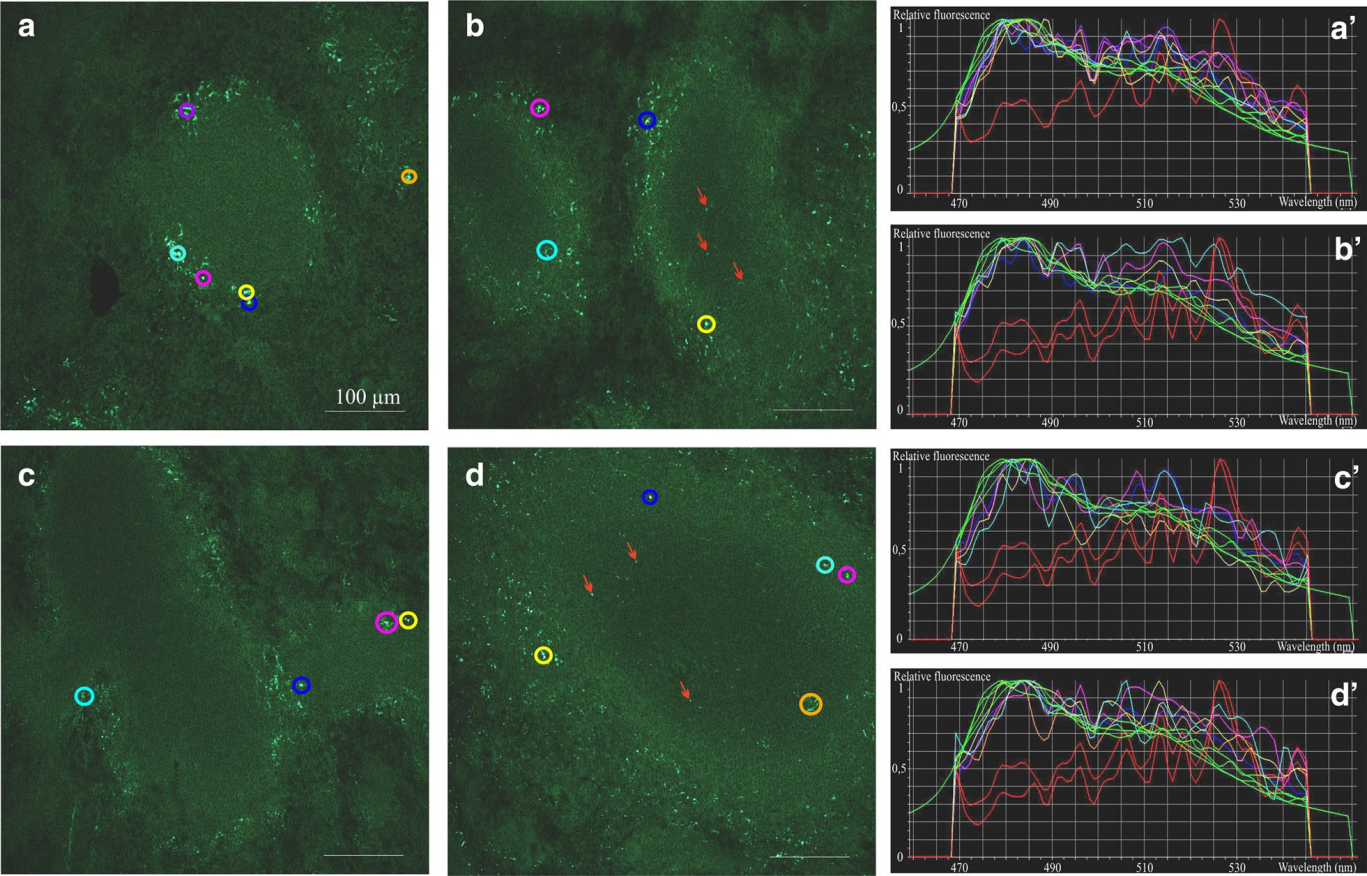
Distribution and accumulation of PS nanoparticles in the spleen. Spectral images of mouse spleen sections after a single intravenous injection of carboxylated (**a**,** b**) or PEGylated (**c**,** d**) polystyrene nanoparticles, 5 min (**a**,** c**) and 4 days (**b**,** d**) after exposure. *Red arrows* indicate translocated particles to the white pulp during the 4-day after exposure period.** a′**–**d′**: spectrum profiles of ROIs in the corresponding images. The spectrum of each ROI is marked with the *same color* as it is delineated in the microscopic image. *Green curves* represent particle fluorescence (positive controls); *red curves* represent tissue autofluorescence (negative controls). *Scale bars* 100 µm

**Fig. 7:**
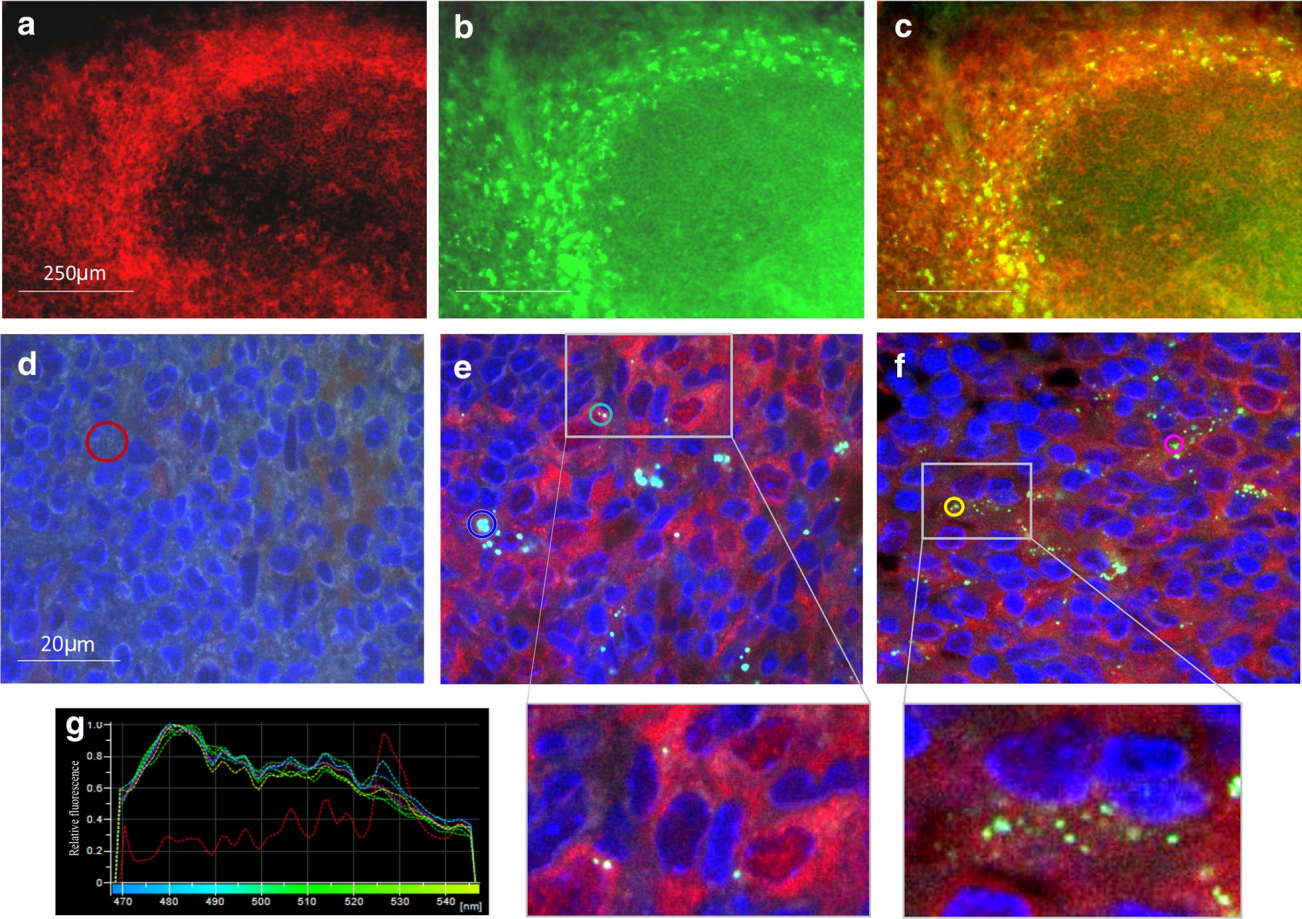
PS-NPs are associated with the marginal zone monocytes/macrophages, identified by Iba-1 staining. Immunohistochemical staining for the spleen macrophages, stained with anti-Iba-1 antibody (**a** *red*), and carboxylated PS-NPs (**b** *green*) 4 days after a single intravenous injection of nanoparticles. Merged image (**c**) shows that residual nanoparticles (*green*) are co-localized with Iba-positivity of marginal zone macrophages (*red*). *Scale bars* 250 µm. Confocal images of spleen sections from non-treated (**d**) or PS-PEG-injected (**e**,** f**) mice. Samples were collected 5 min (**e**) or 4 days (**f**) after exposure to PS- PEG, and were stained for Iba-1. The sample from non-treated animal (**d**) serves also as staining control. Enlarged areas of the boxed regions in** e** and** f**, show the presence of PS-PEG NPs in Iba-1 positive phagocytotic cells.** g** The spectra of ROIs indicated the presence of PS-PEG NPs on images of both, 5-min and 4-day samples. The spectrum of each ROI is marked with the *same color* as it is delineated in the microscopic image. Fluorescence spectra of ROIs were compared against spectrum of tissue autofluorescence (*red curves* as negative controls) and particle fluorescence (*green curves* as positive controls). *Scale bars* 20 µm

In the kidney, both PS-COOH and PS-PEG NPs were present in the glomeruli and also in the interstitium around the tubuli 5 min after nanoparticle administration (Fig. 4a, c). Numerous NPs were identified in the liver (Fig. 5b) and in the spleen (Fig. 6a, c), regardless of the type of NP surface functionalization. In the spleen, NP distribution was mainly restricted to the marginal zone, enriched in monocytes/macrophages. Immunocytochemical analysis directly demonstrated the presence of both PS-COOH and PS-PEG NPs within the intracellular vesicles of Iba-1-positive phagocytotic cells (Fig. 7).

*Four days after nanoparticle administration*, PS-COOH NPs were completely cleared from the brain and the placenta and PS-PEG NPs were not found in these tissues (Additional file 6). Significant reduction of nanoparticle density was also observed in the kidney. While PS-COOH particles were not found in the kidney sections anymore, a few PS-PEG NPs were still deposited within the glomeruli (Fig. 4b, d). In striking contrast, high densities of both NPs were found in the liver (Fig. 5c, d) and in the spleen (Fig. 6b, d). The regional distribution of polystyrene nanoparticles did not change during the 4-day post-loading period in the liver. Characteristic redistribution of NPs was, however, observed within the spleen: 4-days after the exposure, NPs were identified in the white pulp, regardless of surface functionalization (Fig. 7b, d).

Taken together, spectral imaging fluorescence microscopy was instrumental in characterizing the penetration and accumulation of nanoparticle in different organs. The findings also show that differentially-coated nanoparticles exhibit distinct tissue- and cell-type-specific distribution and clearance.

## Discussion

To investigate the impact of surface chemical characteristics of NPs in particle-distribution in the living body, fluorescently core-labeled polystyrene nanoparticles, with different surface characteristics were used. Polystyrene as a core material was chosen, because of its stability in biological solutions, and because ions or toxic compounds could not dissolve from it [37]. Polystyrene in bulk material is not toxic [38] and our previous in vitro data demonstrated that 50 nm polystyrene particles were not toxic to several mouse neural cell lines, when used at concentrations up to 125 μg/ml [33]. Particle surfaces were functionalized either with -COOH^−^ groups presenting strong negative surface charge and mediating ionic interactions or with hydrophilic and charge neutralizing -PEG chains.

Physicochemical characterization of the particles revealed that the applied PS-COOH and PS-PEG particles could be considered identical in their physicochemical aspects, except for the chemical reactivity of their surfaces. At early time points particles did not show significant hydrodynamic size differences in serum containing physiological solutions. Accordingly, PS-COOH and PS-PEG particles were expected to similarly influence the blood flow conditions at the time of intravenous administration. The DLS measurements demonstrated that particles were stabilized in serum-containing fluids, accordingly large-scale aggregate formation was not expected in the circulation, either. The 50–90 nm particle size-range promised several advantages. Particles were comparable in size with natural assemblies of protein complexes, thus were not expected to block circulation in the applied concentration. On the other hand, particles were big enough not to be excreted rapidly by the kidney, which is known to retain proteins and particles larger than 10 nm [4]. Fluorescence spectrum of particles was identical for the two particles regardless of surface functionalization, and was not affected by different environmental conditions, ranging from the dry state to protein containing solutions.

Taken together, their stable spectra, identical hydrodynamic size, and the expected similar effect on flow conditions, PS-COOH and PS-PEG nanoparticles represented ideal tools to investigate the effect of distinct surface characteristics on in vivo accumulation.

Visualization of fluorescent nanoparticles even with high-resolution confocal microscopy is hindered by the size of nanoparticles and by the high autofluorescence of biological samples [12]. The intensity of particle fluorescence depends on the amount of fluorescent dye or on the properties of the core material. In physiological experimentation the use of potentially inert particles labelled with non-toxic signaling compounds is critically important. Therefore, interest was turned to polymer nanoparticles which encapsulate fluorescent dyes. While the covalent embedding of the dye into the core-material prevents dissolution, the amount of “in-core” dye is limited and hinders the fluorescence detection.

In the present study, spectral imaging fluorescence microscopy was used to overcome the limitations caused by high autofluorescence and low particle fluorescence. Spectral image acquisition combined with spectrum analysis could reliably detect fluorescent polystyrene nanoparticles at the tissue- and cellular level.

The distribution of PS-COOH and PS-PEG nanoparticles was investigated at two time points, 5 min and 4 days after particle loading. Initial distribution of particles could not be revealed by histological methods because of the high heart rate (300–800 beats/min) and 20–45 µl stroke volume of mice resulting in an average 20 ml/min cardiac output [39]. The 5 min exposure allowed investigating short-term tissue distribution of particles after a single injection and comparison to a longer term (4 day) distribution.

Five minutes after NP-injection, significantly more PS-COOH then PS-PEG particles were found at the walls of brain vessels and in the placenta. As it was expected [32, 33, 40], PEGylation inhibited the attachment of particles to biological interfaces, while PS-COOH particles could interact with vessel walls and cell surfaces. On the other hand, PEGylation could not prevent the active uptake of PS-PEG NPs by professional macrophages [41, 42]. We found a remarkable accumulation of both PS-COOH and PS-PEG nanoparticles in the kidney, liver and the spleen, e.g. in organs responsible for elimination of particulate polluting agents from the circulation.

Particles were never seen in embryonic tissues indicating a proper placental barrier function against polystyrene nanoparticles, as it was shown also for human placenta [43].

After 4 days, the particles were completely cleared from the vessels of the brain and the placenta, but were accumulated in the liver and the spleen. The presented study did not aim and allow analyzing the renal clearance of the particles. The persisting presence of PS-PEG particles in the glomeruli, however, suggested that particles were accumulated by intraglomerular mesangial cells known to phagocytose contaminating particles, mesangial matrix material and cell debris [44]. In the spleen, several particles were translocated from the marginal zones into the white pulp during the 4-day post-injection period. Our observations on the storage and translocation of these nanoparticles are worthy of further consideration, since they may suggest a method through which compounds on particle surfaces can be presented to the lymphocytes of the marginal zone and the white-pulp [45] and thus manipulate the initiation of adaptive immune responses. Regarding the large-scale material adsorption by NP surfaces including LPS contamination [33], the inflammation-initiating effects of “harmless” NPs should not be neglected.

The observations indicate that middle-sized (50–90 nm) non-biodegradable nanoparticles which are captured by macrophages in the spleen and the liver or even in the kidney interstitium, cannot be easily removed from the body. Regarding the fact that material of dying cells will be ingested by neighboring phagocytes, the question can be raised whether such particles can be cleared at all. Long-term accumulation and limited clearance may cause problems if repeated nanoparticle loading is considered, even if the acute single dose of potentially “harmless” particles is low. Considering the continuous growth of the production and use of nanoparticles, together with the consequent increase in environmental nanoparticle pollution, the long-term fate of nanoparticles in exposed organisms needs further thorough studies. These concerns stress the importance of high-throughput imaging modalities which help to perform distribution studies of tissue- and cell-type-specific NP accumulation. Spectral imaging fluorescence microscopy provides technological tools to monitor the penetration, distribution and accumulation of fluorescently labeled NPs in various tissues with high accuracy and in a time-frame which cannot be achieved with electron microscopy.

## Conclusions

In this work, the in vivo distribution of 50–90 nm polystyrene nanoparticles with distinct surface characteristics was investigated using spectral imaging fluorescence microscopy. Spectral imaging combined with post hoc spectrum analysis allowed visualizing nanoparticles in various tissues and helped to overcome the limitations caused by the high autofluorescence of native tissues. As an additional advantage, the method allowed analyzing larger samples in less time, compared to electron microscopic approaches.

Analyses of the body-distribution of carboxylated or PEGylated polystyrene NPs indicated proper barrier-functions for the blood–brain-barrier and the placenta. While NPs were stuck in the brain vessels and the lacunas of the placenta, barriers completely prevented the penetration of particles into the brain parenchyma and embryonic tissues. Particles with both surface functionalization were accumulated in the reticuloendothelial organs. While PEGylation reduced short-term attachment of particles to vessel walls, it did not prevent accumulation in the liver and the spleen or in the intraglomerular mesangium.

The accumulation and long-term storage of nanoparticles in the reticuloendothelial systems rise important questions on the long-term health-risk even of otherwise non-toxic particles.

## Methods

### Characterization of nanoparticles

Nominal size of 50–70 nm carboxylated and PEGylated (Mw_PEG_ = 300 g/mol) nanoparticles made from “Yellow” fluorochrome-labelled polystyrene, were obtained from Spherotech Inc. (Lake Forest, IL, USA). (Fluorescence spectrum provided by the manufacturer for these “yellow” particles [46]). Concentration of the stock suspensions was 10 mg/ml.

Size and zeta-potential of nanoparticles were measured by ZetasizerNano ZS90 (Horiba Instruments Inc., Irvine, CA). For dynamic light scattering analysis 0.01 mg/ml nanoparticle suspensions were measured using a 633 nm He–Ne laser. Zeta-potential measurements were carried out at 25 °C using a folded capillary cell (DTS1070, Malvern Instruments, Worcestershire, United Kingdom).

Size and shape of nanoparticles were confirmed by transmission electron microscopy (JEOL JEM 1010, JEOL Ltd., Tokyo, Japan) at 80 keV. For TEM analysis, 3 µl aliquots of 0.01 mg/ml nanoparticle suspensions in distilled water were transferred to and dried on 200 mesh copper grids with carbon film.

Particles were stored in sterile MilliQ water and were used within 2–3 months after synthesis.

Raw data of characterization experiments is shown in the Additional file 1.

### Aggregation and protein adsorption of particles in biological solutions

Increase in particle diameter was measured by DLS. Nanoparticle aggregation was monitored in inorganic or biological environments, including solutions used during particle handling and solutions that mimic the characteristics of body fluids. 1 mg/ml carboxylated and PEGylated particle preparations were made by 1:10 dilution of stock suspensions with distilled water. The suspensions were further diluted 1:10 with PBS (pH 7.4), DMEM cell culture medium (Sigma-Aldrich, St. Louis, MO, USA) or DMEM supplemented with 10 % fetal bovine serum (Invitrogen/Gibco, Carlsbad, CA, USA). After 0, 4, 24 or 96-h incubation at 37 °C, particle preparations were diluted in 1:10 with distilled water and the hydrodynamic diameter of particles was measured by DLS.

Proteins adsorbed by particles in 10 % fetal bovine serum containing minimum essential medium (FBS-MEM; Sigma-Aldrich, St. Louis, MO, USA) were analyzed by SDS-PAGE (Sodium dodecyl sulfate polyacrylamide gel electrophoresis). After 1 or 24-h incubation in 10 % FBS-MEM nanoparticles were sedimented by centrifugation (45 min at 20,000×*g*) and were washed with PBS to remove non-bound proteins. Washed NPs were resuspended in Laemmli buffer containing 1 % (w/v) sodium dodecyl sulfate, and loaded onto 10 % polyacrylamide gel. Gel electrophoresis was performed at 130 V for about 60 min. The gels were stained with silver staining 33 kit (Cosmobio Ltd., Tokyo, Japan), according to the manufacturer’s instructions.

### In vivo experiments

Animal experiments were conducted with the approval of the Animal Care Committee of the Institute of Experimental Medicine of Hungarian Academy of Sciences and according to the official license (No.: 22.1/353/3/2011; exp. date: 4/7/2016) issued by National Food Chain Safety Office (http://www.NEBIH.gov.hu), Hungary.

Male mice (aged 25–30 days) and pregnant female mice on the 10th to 15th post conception days were anesthetized with a mixture of ketamine (CP-Pharma mbH, Burgdorf, Germany) and xylazine (CEVA-PHYLAXIA, Budapest, Hungary), 100 and 10 µg/g bodyweight, respectively. Nanoparticle stock suspensions (10 mg/ml) were diluted 1:30 in PBS and dispersed by sonication. Under proper anesthesia, 7 µl/g bodyweight aliquots of carboxylated (n_COOH(male)_ = 6, n_COOH(pregnant female)_ = 6) or PEGylated (n_PEG(male)_ = 6, n_PEG(pregnant female)_ = 6) nanoparticle suspensions were introduced into the tail vein. Animals were sacrificed by overdose of anesthetics after a 5-min or 4-day exposure to the single-injection loading. Various organs including brain, liver, kidney, spleen as well as placenta and embryos were removed and fixed with paraformaldehyde (8 w/v % in PBS) for 24 h at 4 °C. Organs and embryos were collected from animals not exposed to nanoparticles, as controls (n_control(male)_ = 3, n_control(pregnant female)_ = 3).

### Microscopic evaluation

For microscopic evaluation, 30 or 60 µm thick vibratome sections (VT1000S, Leica, Wetzlar, Germany) were made from fixed organs.

For immunocytochemical staining, the sections were permeabilized and non-specific antibody binding was blocked by incubating the sections in PBS containing 10 % FBS and 0.1 % Triton-X for 2 h. Primary antibodies, anti-Iba-1 goat polyclonal antibodies and anti-claudin V rabbit polyclonal antibodies (Abcam; Cambridge, UK) were diluted in 1–1000 with PBS-FBS, and the sections were incubated at 4 °C, overnight. After incubation, the cells were washed three times (15 min each) with PBS and incubated with alexa-594 conjugated secondary antibodies (1:1000; Molecular Probes, Invitrogen, Carlsbad, CA, USA) for 1 h. Slices were mounted with mowiol (Sigma-Aldrich, Budapest, Hungary) containing 10 μg/ml bisbenzimide (Sigma-Aldrich) for nuclear staining.

Sections were analyzed with a Zeiss Axiovert 200 M fluorescence microscope (Zeiss, Jena, Germany) and a Nikon A1R Confocal Laser Microscope System equipped with a spectral detector unit (Nikon, Shinjuku, Tokyo, Japan).

### Optimized detection of particles by spectral imaging fluorescence microscopy

Fluorescence spectra of NPs were determined in dry and in buffer-dispersed particle preparations, as well as in contact with fixed tissue sections from control animals by spectral imaging fluorescence microscopy. The extrinsic spectra emitted by the particles in contact with the corresponding tissue section from control animals were used as positive controls. For negative control, the intrinsic autofluorescence spectra of corresponding sections of control organs were used (Additional file 3).

For spectral evaluation 457 nm argon ion laser was used as excitation source, and the emitted light was detected by the spectral detector unit from 468 to 548 nm, with a spectral resolution of 2.5 nm. In order to record continuous spectrum, a 20/80 beam splitter (BS20/80) with continuous transmission was used instead of a paired dichronic mirror arrangement.

Regions of interest (ROI) were delineated and analyzed in comparison with corresponding sections of nanoparticle-treated and non-treated organs. The photocurrent intensities detected in the samples at different wavelengths (emission spectra in organs of treated animals) were plotted against the tissue autofluorescence spectra (negative control) and the spectra of nanoparticles seeded on control-tissue (positive control).

## Additional files

10.1186/s12951-016-0210-0 Raw results of characterization experiments. PS-NPs were incubated with inorganic or biological solutions. After 5-min, 4, 24 or 96 h incubation size distribution and zeta-potential measurements were carried out by a ZetasizerNano ZS90.
10.1186/s12951-016-0210-0 Excitation of fluorophore labelled polystyrene nanoparticles. Nanoparticles were excited at 404, 457, 476 nm wavelengths to determine optimal excitation settings. Highest intensity of emitted light was reached when samples were excited at 457 nm.
10.1186/s12951-016-0210-0 Intrinsic fluorescence of non-treated tissue sections. Spectral images showing autofluorescence of non-treated brain (A), placenta (B), kidney (C) and spleen (D) sections. Green curves represent particle fluorescence (positive controls); the red curve represents the autofluorescence. The autofluorescence spectra were used as negative controls for post hoc spectral identification of PS-NPs in the corresponding tissues. A’, B’, C’, D’: spectrum profiles of ROIs in the corresponding images. The spectrum of each ROI is marked with the same color as it is delineated in the microscopic image.
10.1186/s12951-016-0210-0 Polystyrene NPs in brain vessels 5 min after systemic exposure. Fluorescence images of sections made from brain of PS-PEG (A) or PS-COOH (B) injected adult mice. Animals were sacrificed 5 min after intravenous injection. Sections were stained for Claudin V (red); cell nuclei are shown in blue. Scale bars: 50 µm.
10.1186/s12951-016-0210-0 Embryonic tissues were free from nanoparticles 5 min after maternal NP-administration. Sections made from mouse embryonic (E 15) forebrain cortex (A, B, C) and liver (D, E) 5 min after the injection of carboxylated PS nanoparticles into the tail vein of the mother. Cell nuclei are stained with bisbenzimide (blue). Representative spectrum images (C, E) and spectrum profiles (C’, E’) showed no particles in the embryonic brain or liver tissues. Scale bars: 50 µm.
10.1186/s12951-016-0210-0 PS-NP cleared from the brain and the placenta within the 4-day post-injection period. Spectral images of sections of mouse brain (A, B) and placenta (C, D) 4 days after injection of PS-COOH (A, C) or PS-PEG (B, D) nanoparticles into the tail vein of adult mice. A’, B’, C’, D’: spectrum profiles of ROIs in the corresponding images. The spectrum of each ROI is marked with the same color as it is delineated in the microscopic image. Green curves represent particle fluorescence (positive controls); red curve represents tissue autofluorescence (negative control). Scale bars: 50 µm.

## Abbreviations

BSA: bovine serum albumin
DLS: dynamic light scattering
DMEM: Dulbecco’s modified Eagle’s cell culture medium
FBS: fetal bovine serum
MEM: minimum essential medium
NP: nanoparticles
PBS: phosphate buffered saline
PEG: polyethylene glycol
PS-NP: polystyrene nanoparticles
PS-COOH: carboxylated polystyrene nanoparticles
PS-PEG: PEGylated polystyrene nanoparticles
ROI: regions of interest
SDS-PAGE: sodium dodecyl sulfate polyacrylamide gel electrophoresis
TEM: transmission electron microscopy
