# Supplementary figures and images for: Enhanced detection with spectral imaging fluorescence microscopy reveals tissue- and cell-type-specific compartmentalization of surface-modified polystyrene nanoparticles
Source: J Nanobiotechnology. 2016 Jul 7;14:55. doi: 10.1186/s12951-016-0210-0 (PMC4936314; doi:10.1186/s12951-016-0210-0)

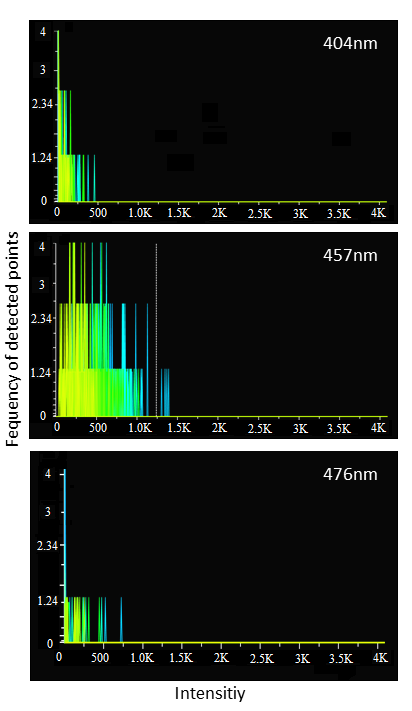

Supplement: Supplementary file 2 — 10.1186/s12951-016-0210-0 Excitation of fluorophore labelled polystyrene nanoparticles. Nanoparticles were excited at 404, 457, 476 nm wavelengths to determine optimal excitation settings. Highest intensity of emitted light was reached when samples were excited at 457 nm. [file 12951_2016_210_MOESM2_ESM.png]

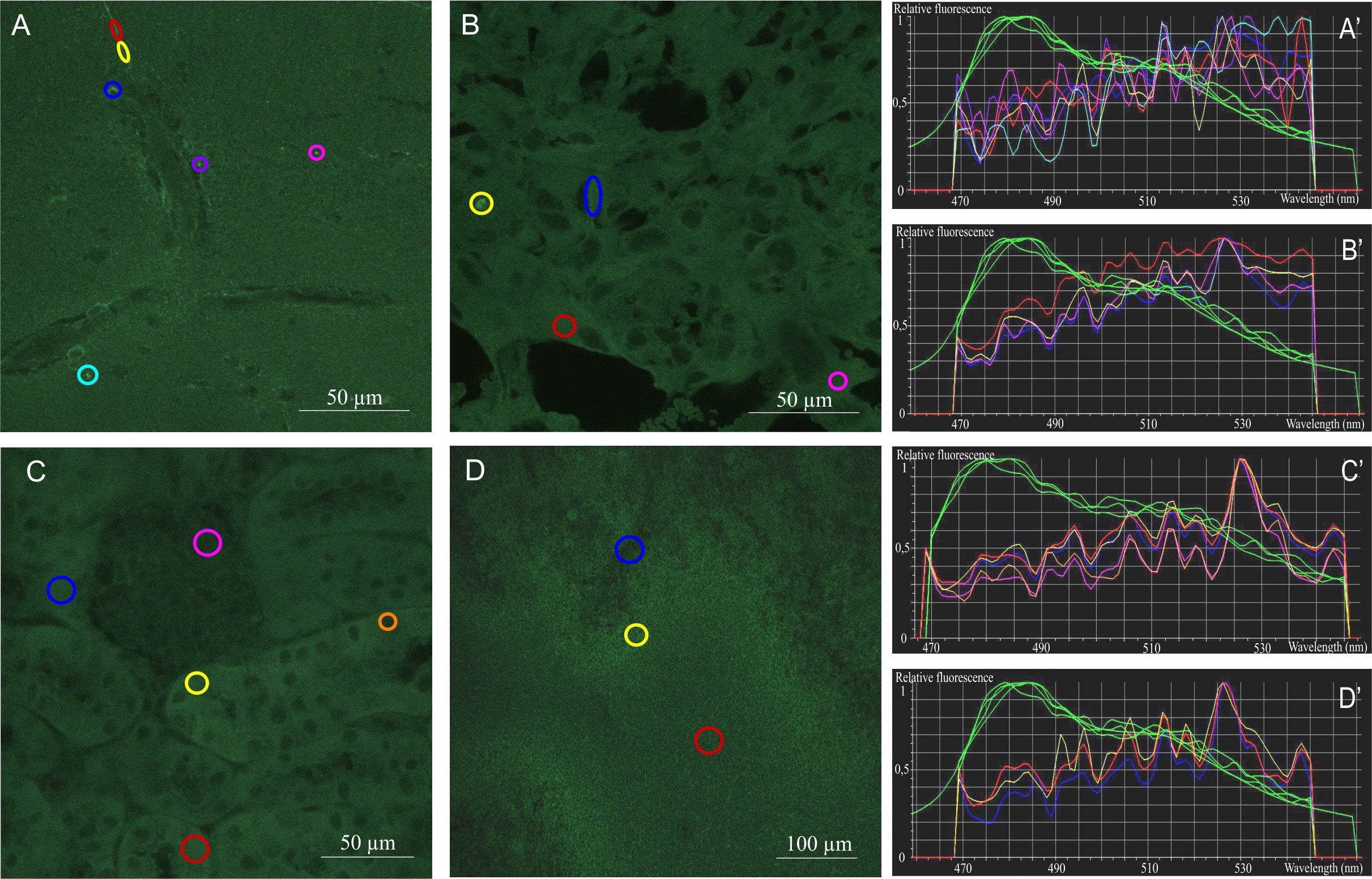

Supplement: Supplementary file 3 — 10.1186/s12951-016-0210-0 Intrinsic fluorescence of non-treated tissue sections. Spectral images showing autofluorescence of non-treated brain (A), placenta (B), kidney (C) and spleen (D) sections. Green curves represent particle fluorescence (positive controls); the red curve represents the autofluorescence. The autofluorescence spectra were used as negative controls for post hoc spectral identification of PS-NPs in the corresponding tissues. A’, B’, C’, D’: spectrum profiles of ROIs in the corresponding images. The spectrum of each ROI is marked with the same color as it is delineated in the microscopic image. [file 12951_2016_210_MOESM3_ESM.tif]

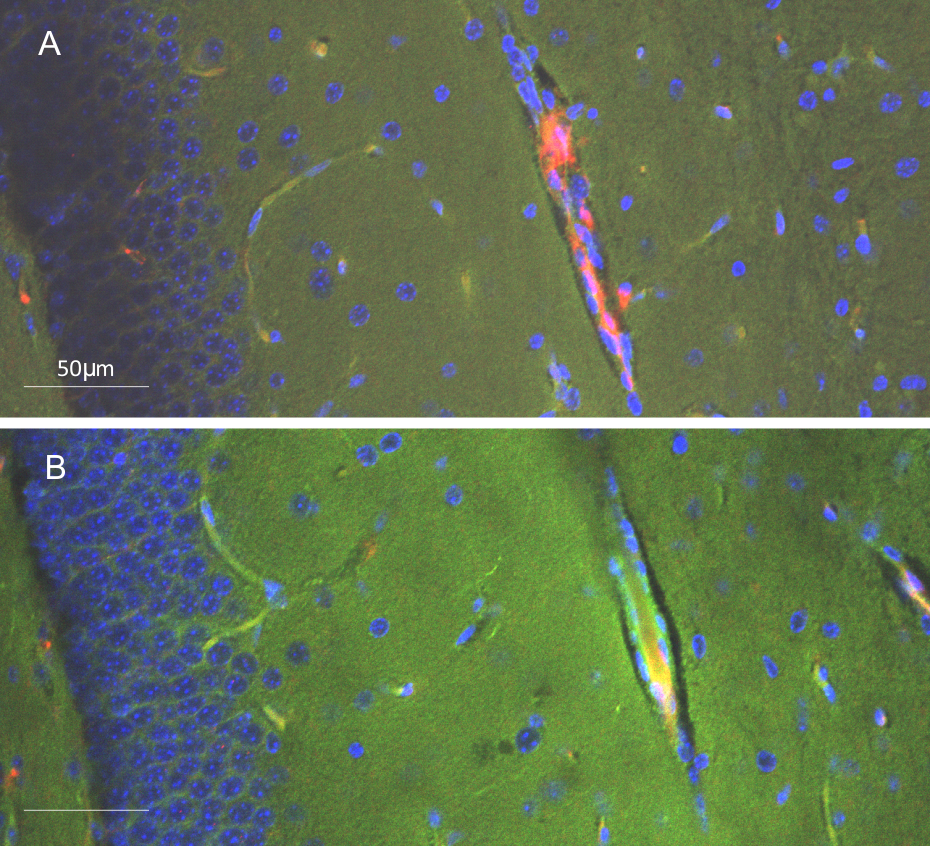

Supplement: Supplementary file 4 — 10.1186/s12951-016-0210-0 Polystyrene NPs in brain vessels 5 min after systemic exposure. Fluorescence images of sections made from brain of PS-PEG (A) or PS-COOH (B) injected adult mice. Animals were sacrificed 5 min after intravenous injection. Sections were stained for Claudin V (red); cell nuclei are shown in blue. Scale bars: 50 µm. [file 12951_2016_210_MOESM4_ESM.tif]

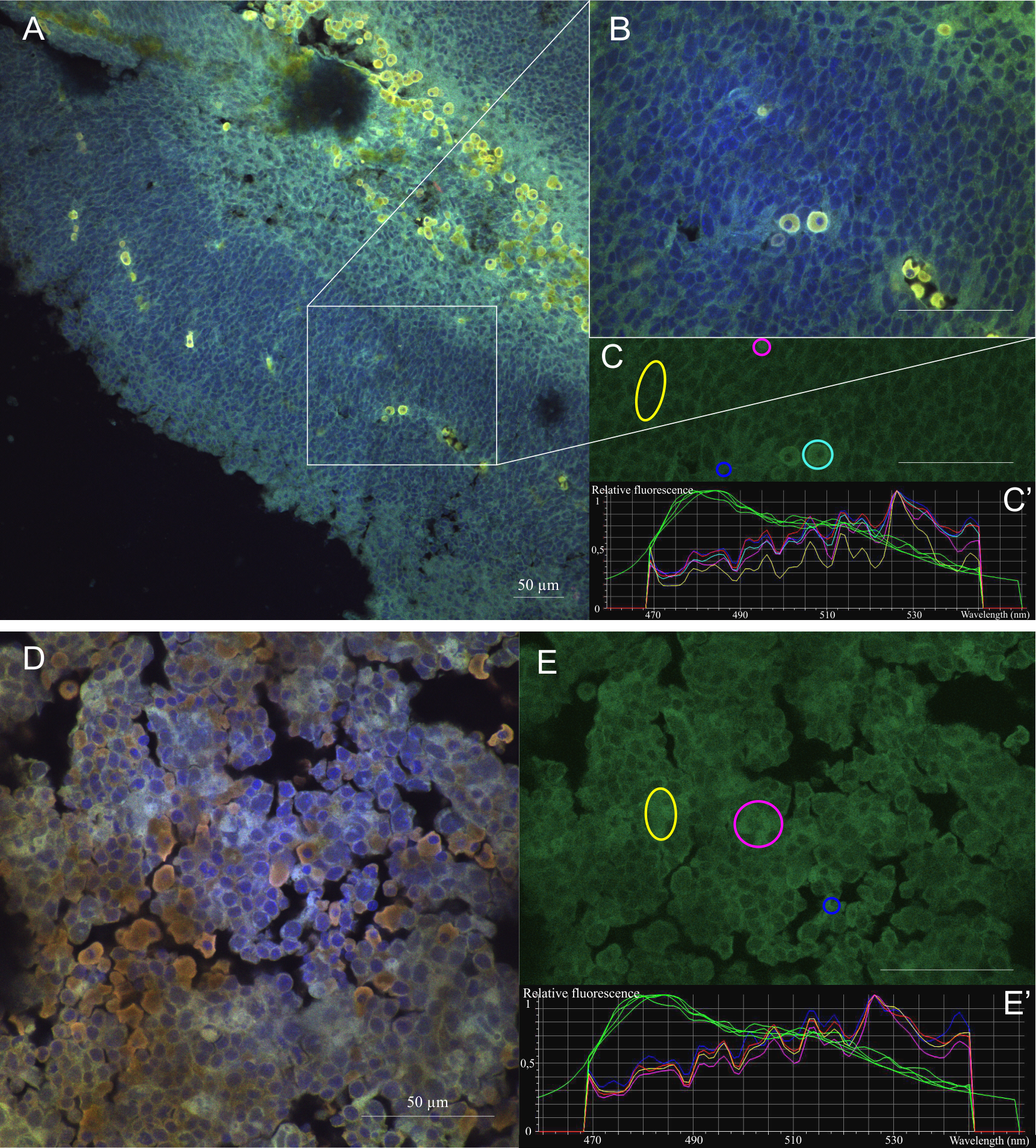

Supplement: Supplementary file 5 — 10.1186/s12951-016-0210-0 Embryonic tissues were free from nanoparticles 5 min after maternal NP-administration. Sections made from mouse embryonic (E 15) forebrain cortex (A, B, C) and liver (D, E) 5 min after the injection of carboxylated PS nanoparticles into the tail vein of the mother. Cell nuclei are stained with bisbenzimide (blue). Representative spectrum images (C, E) and spectrum profiles (C’, E’) showed no particles in the embryonic brain or liver tissues. Scale bars: 50 µm. [file 12951_2016_210_MOESM5_ESM.tif]

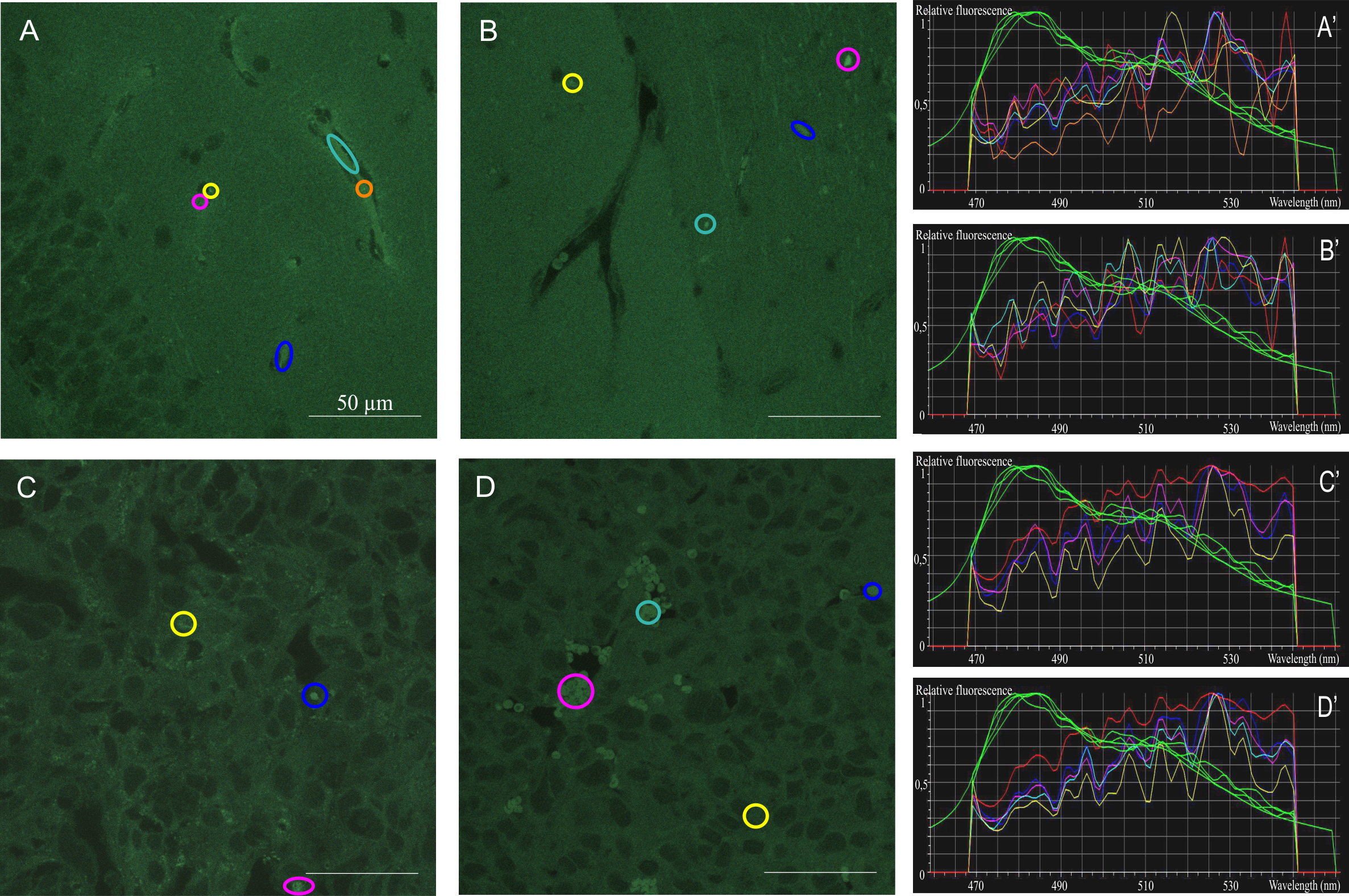

Supplement: Supplementary file 6 — 10.1186/s12951-016-0210-0 PS-NP cleared from the brain and the placenta within the 4-day post-injection period. Spectral images of sections of mouse brain (A, B) and placenta (C, D) 4 days after injection of PS-COOH (A, C) or PS-PEG (B, D) nanoparticles into the tail vein of adult mice. A’, B’, C’, D’: spectrum profiles of ROIs in the corresponding images. The spectrum of each ROI is marked with the same color as it is delineated in the microscopic image. Green curves represent particle fluorescence (positive controls); red curve represents tissue autofluorescence (negative control). Scale bars: 50 µm. [file 12951_2016_210_MOESM6_ESM.tif]
